# Effects of Plant Polysaccharides on Growth Performance, Blood Biochemical Indices, Intestinal Antioxidant and Enzyme Activities, and Microbial Diversity in Early-Weaned Squabs

**DOI:** 10.3390/ani16121785

**Published:** 2026-06-09

**Authors:** Jie Ren, Yuanhao Li, Huiguo Yang, Haiying Li, Xiaobin Li, Xiaoyu Zhao, Yafei Liang, Mingcong Ding, Haiying He, Aikemu Mamaitijiang, Honglei Sun, Jiajia Liu

**Affiliations:** 1 College of Animal Science, Xinjiang Agricultural University, Urumqi 830052, China; 15133391369@163.com (J.R.); 17769614800@163.com (Y.L.); 13629439845@163.com (H.L.); lxb262819@163.com (X.L.); vz_zxy@163.com (X.Z.); 15329625726@163.com (M.D.); sunhonglei0805@163.com (H.S.); 2 Institute of Animal Husbandry, Xinjiang Academy of Animal Husbandry, Urumqi 830011, China; 13809951421@163.com (H.Y.); m13934057294@163.com (Y.L.); 15132311203@163.com (H.H.); 3 Moyu Blue Sea Pigeon Industry Co., Ltd., Hetian 848100, China; 13899912402@163.com

**Keywords:** Astragalus polysaccharides, Glycyrrhiza polysaccharides, blood biochemistry, microbial diversity, intestinal antioxidant capacity

## Abstract

Plant polysaccharides, such as Astragalus polysaccharide (APS) and Glycyrrhiza polysaccharide (GPS), have potential as functional feed additives. This study investigated the effects of dietary APS and GPS on growth-related traits, serum biochemical and immune indices, antioxidant capacity, intestinal health, and microbial diversity in early-weaned squabs. A total of 192 15-day-old Silver King squabs were randomly divided into four groups: the control group (CK), the 800 mg/kg APS group, the 450 mg/kg GPS group, and the APS + GPS combination group (AG group), with 12 replicates per group and 4 squabs per replicate. The experiment lasted for 28 days. The results showed that final body weight tended to be higher in the APS, GPS, and AG groups, whereas breast width and breast depth were significantly increased in the GPS and AG groups (*p* < 0.01). The GPS and AG groups exhibited increased serum immunoglobulin A (IgA; *p* < 0.05) and immunoglobulin G (IgG; *p* < 0.01) levels, as well as reduced levels of pro-inflammatory cytokines, including interleukin-6 (IL-6), interleukin-1β (IL-1β), and tumor necrosis factor-α (TNF-α; *p* < 0.01). All treatments increased serum total antioxidant capacity (T-AOC; *p* < 0.01), while the AG group reduced malondialdehyde (MDA) levels and increased total superoxide dismutase (T-SOD) and glutathione peroxidase (GSH-Px) activities (*p* < 0.01). Duodenal and jejunal T-AOC increased in all treatment groups (*p* < 0.01), and APS and AG increased ileal T-AOC (*p* < 0.01). However, intestinal MDA concentrations increased in several segments, indicating a complex and segment-specific oxidative response. The AG group also increased jejunal lipase activities (*p* < 0.05). Microbiome analysis suggested that *Helicobacter* was correlated with immune-related indicators, while *Lactobacillus* was identified as an important core genus in the microbial co-occurrence network. These findings suggest that dietary APS and GPS may regulate immune function, oxidative–antioxidant responses, intestinal function, and gut microbial composition, thereby supporting physiological adaptation in early-weaned squabs.

## Simple Summary

Early weaning is commonly used in pigeon farming to improve production efficiency. However, it often causes stress in squabs, leading to poor growth, digestive problems, weakened immunity, and imbalance of gut bacteria. In this study, we added plant-derived polysaccharides from Astragalus (APS) and Glycyrrhiza (GPS), either alone or in combination, to the diet of early-weaned squabs. The results suggested that APS and GPS, especially their combination, may help regulate immune and antioxidant responses, support intestinal structure and digestive function, and modulate the gut microbial community. These findings indicate that APS and GPS may contribute to physiological adaptation and intestinal health in early-weaned squabs.

## 1. Introduction

In pigeon meat production, early weaning effectively accelerates the turnover of breeding pigeons and enhances breeding efficiency [1]. During the transition from parental feeding to artificial feeding, squabs often encounter multiple challenges, including sudden changes in nutrient sources, environmental stress, and immature intestinal development, which can result in growth retardation, damage to the intestinal mucosal barrier, exacerbated inflammatory responses, and disruption of the intestinal flora [2]. Previous studies have demonstrated that early weaning markedly reduces squab growth performance, induces atrophy of ileal villi, increases intestinal permeability, alters the expression of tight junction-related genes, and disturbs the structure of the intestinal flora, which is considered the primary factor contributing to the decline in production performance post-weaning [3].

Therefore, the selection of safe and effective green feed additives to alleviate weaning stress and maintain intestinal homeostasis has become an important research focus in pigeon nutrition regulation. Liang et al. reported that plant polysaccharides, as natural macromolecules characterized by wide availability, low toxicity, and diverse biological activities, possess antioxidant, anti-inflammatory, immunoregulatory, and intestinal barrier–enhancing functions [4]. Previous studies have shown that plant-derived polysaccharides not only improve intestinal morphology and reduce intestinal permeability in poultry, but also regulate the intestinal microbiota structure, promote nutrient digestion and absorption, and, to a certain extent, enhance growth performance and antioxidant capacity [5]. In poultry and other early-weaned animals, alfalfa polysaccharides have been shown to improve broiler growth performance by modulating the intestinal microbiota, promoting intestinal development, and improving overall health [6], whereas licorice polysaccharides increase the average daily gain (ADG) of weaned piglets and improve digestive enzyme activity, intestinal barrier function, and intestinal flora structure [7]. These findings indicate that plant polysaccharides exhibit favorable effects in alleviating weaning stress and maintaining intestinal health. In pigeon rearing, numerous studies related to polysaccharides have demonstrated that chitosan oligosaccharides and their synbiotics with *Clostridium butyricum* improve intestinal morphology, digestive enzyme activity, antioxidant status, and intestinal flora diversity in early-weaned squabs [8]. Sun et al. further demonstrated that Cordyceps militaris polysaccharides improve intestinal tissue morphology and regulate the ileal microbiota structure in squabs, thereby promoting intestinal health [9]. However, compared with chitosan oligosaccharides and fungal polysaccharides, research on plant polysaccharides in early-weaned squabs remains relatively limited, particularly regarding their coordinated regulatory effects on growth performance, blood biochemical indices, intestinal antioxidant status, digestive enzyme activity, and intestinal flora diversity. Therefore, investigation of the effects of plant polysaccharides on growth and intestinal health in early-weaned squabs not only contributes to a deeper understanding of their mechanisms in alleviating weaning stress, but also provides a theoretical basis for the development of green feed additives and antibiotic-free healthy pigeon production [5].

## 2. Materials and Methods

### 2.1. Ethical Considerations

All animal care and experimental procedures adhered to the Guidelines for the Care and Use of Experimental Animals in China and were approved by the Experimental Animal Ethics Committee of the Institute of Animal Husbandry, Academy of Animal Husbandry, Xinjiang Uygur Autonomous Region (project license number: 202602).

### 2.2. Additive

APS and GPS used in the present study were obtained from Lyman Xiangrong Biotechnology Co., Ltd. (Xi’an, China), with purities of 68% and 40%, respectively. The CK group received a basal diet without polysaccharide supplementation, whereas the APS, GPS, and AG groups received the basal diet supplemented with 800 mg/kg APS, 450 mg/kg GPS, and a combination of 800 mg/kg APS and 450 mg/kg GPS, respectively. The supplementation levels were determined according to the manufacturer’s recommendations and previous studies concerning the application of Astragalus polysaccharides and Glycyrrhiza polysaccharides in animal nutrition [4,7].

### 2.3. Animal and Experimental Design

The animal experiment was performed at Moyu Blue Sea Pigeon Industry Development Co., Ltd. (Hotan, Xinjiang, China). A total of 192 healthy 15-day-old early-weaned Silver King squabs were randomly allocated to four dietary treatments, namely the CK, APS, GPS, and AG groups, with 12 replicates per treatment and four squabs per replicate, resulting in 48 squabs per treatment. The experimental period lasted 28 days. Squabs were housed in cages measuring 45 cm × 50 cm × 60 cm and were provided ad libitum access to feed and water. A lighting schedule of 16 h of light per day was maintained, and room temperature was controlled at 15 ± 5 °C. Identical environmental and feeding conditions were applied to all groups, with dietary supplementation strategy representing the only experimental variation. For growth performance evaluation, including body weight, ADG, average daily feed intake (ADFI), and feed-to-gain ratio (F/G), the cage served as the experimental unit, with 12 replicate cages per treatment and four squabs per cage. For analyses of serum biochemical parameters, immune indices, intestinal antioxidant parameters, digestive enzyme activities, intestinal morphology, and microbiota composition, individual squabs were treated as independent biological replicates. At day 28, one squab was randomly selected from each replicate cage, resulting in 12 biological replicates per treatment group (n = 12). Ileal samples used for microbiota analysis were collected individually and sequenced independently without pooling prior to DNA extraction. Throughout the experiment, all squabs received a basal diet formulated to satisfy the nutritional requirements of meat pigeons. The composition and nutrient levels of the basal diet are presented in Table 1.

### 2.4. Measurement Indicators and Methods

#### 2.4.1. Growth Performance

Body weights of fasting squabs were measured at 0, 7, 14, 21, and 28 days of age using an electronic balance (maximum weighing capacity: 1 kg; Yueping Instrument Co., Ltd., Shanghai, China). Feed offered and residual feed were recorded daily throughout the experimental period. Average body weight, ADG, ADFI, and F/G were subsequently calculated for squabs in each group at different ages.

#### 2.4.2. Body Dimensions

Body size traits of squabs were measured at 0 and 28 days of age. Body oblique length, keel length, tibia length, shank circumference, breast width, and breast depth were determined using a flexible measuring tape and a vernier caliper (range: 0–150 mm; precision: 0.02 mm). The flexible measuring tape was purchased from Huitong Measuring Tool Co., Ltd. (Ningbo, China), and the digital vernier caliper (0–150 mm, 0.02 mm) was provided by Harbin Measuring & Cutting Tool Group Co., Ltd. (Harbin, China). The total gain of each morphometric trait from day 0 to day 28 was subsequently calculated. All measurements were conducted according to the standard terminology and measurement methods for poultry production performance.

#### 2.4.3. Serum Parameters

On day 28, following 4 h of feed deprivation, 12 squabs were randomly selected from each group, resulting in a total of 48 individuals. A 5 mL blood sample was collected from the wing vein of each bird. Blood samples were centrifuged at 2500 r/min for 10 min using a TDL-5-A bench-top centrifuge (Anke Instrument Co., Ltd., Shanghai, China). The resulting supernatants were aliquoted into 1.5 mL centrifuge tubes and temporarily stored at −20 °C in a DW-25L262 low-temperature refrigerator (Haier Biomedical Co., Ltd., Qingdao, China). All serum-related indices were analyzed by Beijing Huaying Testing Technology Co., Ltd. (Beijing, China). The detected parameters were classified into three categories: (1) serum biochemical parameters, including total protein (TP), albumin (ALB), total cholesterol (TC), triglyceride (TG), globulin (GLB), high-density lipoprotein (HDL), low-density lipoprotein (LDL), alanine transaminase (ALT), and aspartate transaminase (AST); (2) serum immune indices, including immunoglobulin A (IgA), immunoglobulin G (IgG), immunoglobulin M (IgM), interleukin-6 (IL-6), interleukin-10 (IL-10), interleukin-1β (IL-1β), and tumor necrosis factor-α (TNF-α); and (3) serum oxidative and antioxidant indices, including total antioxidant capacity (T-AOC), total superoxide dismutase (T-SOD), glutathione peroxidase (GSH-Px) activity, and malondialdehyde (MDA) content.

#### 2.4.4. Intestinal Antioxidation

After slaughter, intestinal tissue samples were collected from 12 squabs in the control group and each of the three experimental groups, resulting in a total of 48 samples, and subsequently homogenized. The samples were thoroughly mixed with physiological saline at a mass-to-volume ratio of 1:9. The homogenates were centrifuged at 3000 rpm for 10 min at 4 °C, and the resulting supernatants were collected for the determination of SOD, GSH-Px, T-AOC, and MDA content. All assays were conducted by Nanjing Jiancheng Bioengineering Institute (Nanjing, China).

#### 2.4.5. Intestinal Enzyme Activity

Subsequently, duodenal and jejunal contents were collected from 12 squabs in the control group and each of the three experimental groups, resulting in a total of 48 samples. All specimens were transferred into cryopreservation tubes, rapidly frozen in liquid nitrogen, and stored at −80 °C for subsequent analysis. The activities of amylase, trypsin, chymotrypsin, and lipase were determined by Nanjing Jiancheng Bioengineering Institute (Nanjing, China).

#### 2.4.6. Morphological Observation of the Intestine

For histological evaluation, 12 squabs per treatment group, with one bird randomly selected from each replicate cage, were collected on day 28, resulting in a total of 48 samples for intestinal morphology analysis. Segments measuring 2–3 cm in length were obtained from the duodenum, jejunum, and ileum of each pigeon, rinsed with physiological saline, and fixed in 4% paraformaldehyde. Intestinal sections were sliced continuously at a thickness of 5 μm using a microtome, stained with hematoxylin and eosin (HE), and subsequently observed and photographed under an optical microscope. Villus height (VH) and crypt depth (CD) were measured using image analysis software (C.V2.4) provided by Servicebio (Wuhan Servicebio Technology Co., Ltd., Wuhan, China). For each intestinal section, three well-oriented villi and their corresponding crypts were selected for measurement, and the average value was used for statistical analysis. The VH-to-CD ratio (V/C ratio) was subsequently calculated.

#### 2.4.7. Gut Microbiota Analysis

Ileal contents were collected on day 28 from 12 squabs randomly selected from each treatment group (n = 12 per treatment; total n = 48). All samples were collected individually and stored at −80 °C until analysis. No pooling procedure was performed, and each sample was independently processed for sequencing analysis. Microbial genomic DNA was extracted from ileal contents, and the V3–V4 hypervariable regions of bacterial 16S ribosomal RNA (16S rRNA) genes were amplified. Sequencing was conducted on the Illumina NovaSeq platform using paired-end 250 bp reads (PE250). Raw sequencing reads were assigned to samples according to barcode sequences. Paired-end reads were merged using FLASH (version 1.2.11), followed by primer trimming and quality filtering with fastp. Chimeric sequences were subsequently removed to obtain effective tags. After quality filtering and chimera removal, the number of effective tags ranged from 51,573 to 104,725 per sample, indicating sufficient sequencing depth for subsequent microbiota analysis. Sequence denoising was conducted using the Divisive Amplicon Denoising Algorithm 2 (DADA2) implemented in Quantitative Insights Into Microbial Ecology 2 (QIIME2), and sequence denoising was conducted using DADA2 implemented in QIIME2, and amplicon sequence variants (ASVs) were generated for downstream analyses. Taxonomic annotation was performed using the classify-sklearn algorithm in QIIME2 based on the SILVA 138.1 database. Rarefaction analysis and Good’s coverage values were used to evaluate sequencing depth and coverage. Alpha diversity, beta diversity, linear discriminant analysis effect size (LEfSe), BugBase phenotype prediction, Phylogenetic Investigation of Communities by Reconstruction of Unobserved States (PICRUSt) functional prediction, and correlation analyses were subsequently conducted.

### 2.5. Data Statistics and Analysis

Raw data were sorted and processed using Microsoft Excel 2021 (Microsoft Corporation, Redmond, WA, USA). Prior to statistical analysis, normality of the data was evaluated using the Shapiro–Wilk test, and homogeneity of variance was assessed using Levene’s test. Data satisfying these assumptions were analyzed using one-way analysis of variance (one-way ANOVA) with IBM SPSS Statistics 27.0 (IBM Corporation, Armonk, NY, USA). Duncan’s multiple range test was used for post hoc comparisons among treatment groups. All experimental data were expressed as mean ± standard deviation (SD). A *p*-value < 0.05 was considered statistically significant, whereas a *p*-value < 0.01 was considered highly significant.

## 3. Results

### 3.1. Effects of Dietary Plant Polysaccharides on Growth Performance of Squabs

The effects of dietary plant polysaccharides on the growth performance of squabs are presented in Table 2. ADFI and ADG exhibited increasing trends in all treatment groups (*p* > 0.05), whereas the F/G showed a decreasing trend (*p* > 0.05).

### 3.2. Effects of Dietary Plant Polysaccharides on Body Measurements of Squabs

The effects of dietary APS and GPS supplementation on body measurements of squabs are summarized in Table 3. At 28 days of age, all experimental groups (APS, GPS, and AG) exhibited significantly greater breast width and breast depth than the control group (CK) (*p* < 0.01). Regarding tibia length, the GPS group showed a significantly greater value than the APS group (*p* < 0.05), whereas no significant difference was observed between the CK and GPS groups. Keel length was significantly greater in the GPS and AG groups than in the CK group (*p* < 0.01). Over the entire 0–28-day period, total gains in breast width and breast depth were significantly higher in all experimental groups than in the CK group (*p* < 0.01). The total gain in tibia length was significantly lower in the APS group than in the GPS group (*p* < 0.05). In addition, the AG group exhibited a significantly greater total gain in body length than the CK group (*p* < 0.05). No significant differences in shank circumference were observed at any time point.

### 3.3. Effects of Dietary Supplementation with Plant Polysaccharides on Serum Biochemical Indices of Squabs

The effects of dietary plant polysaccharide supplementation on serum biochemical indices of squabs are shown in Table 4. No significant differences were observed between the experimental groups and the CK group.

### 3.4. Effects of Dietary Supplementation with Plant Polysaccharides on Serum Immune Indices of Squabs

The effects of dietary plant polysaccharide supplementation on serum immune indices of squabs are presented in Table 5. Regarding immunoglobulins, the AG group showed a significantly higher IgA level than the CK group (*p* < 0.05), whereas the IgG level was markedly increased compared with that in the CK group (*p* < 0.01). Concerning inflammation-related cytokines, the GPS and AG groups exhibited significantly reduced levels of the pro-inflammatory factors IL-6, IL-1β, and TNF-α compared with the CK group (*p* < 0.01), while the level of the anti-inflammatory factor IL-10 was significantly increased (*p* < 0.01).

### 3.5. Effects of Dietary Supplementation with Plant Polysaccharides on Serum Antioxidant Indices of Squabs

The effects of dietary plant polysaccharide supplementation on serum antioxidant capacity in squabs are presented in Table 6. Compared with the CK group, T-AOC was significantly increased in all experimental groups (*p* < 0.01). T-SOD activity was significantly higher in the GPS and AG groups (*p* < 0.01). GSH-Px activity was significantly elevated in the AG group (*p* < 0.01), while MDA content was significantly lower in the AG group compared with the CK group (*p* < 0.01).

### 3.6. Effects of Dietary Supplementation with Plant Polysaccharides on Intestinal Oxidative and Antioxidant Indices of Squabs

The effects of dietary plant polysaccharide supplementation on intestinal antioxidant capacity are shown in Table 7. In the duodenum, T-AOC was significantly increased in the APS, GPS, and AG groups compared with the CK group (*p* < 0.01). T-SOD activity was significantly elevated in the APS group (*p* < 0.05), and MDA content was significantly increased in the APS and AG groups (*p* < 0.01). In the jejunum, T-AOC was significantly higher in the APS, GPS, and AG groups (*p* < 0.01), and MDA content was significantly elevated in the GPS and AG groups (*p* < 0.01). In the ileum, T-AOC was significantly increased in the APS and AG groups (*p* < 0.01), GSH-Px activity was significantly decreased in the APS group (*p* < 0.05), and MDA content was significantly increased in the AG group (*p* < 0.01).

### 3.7. Effects of Dietary Supplementation with Plant Polysaccharides on Intestinal Enzyme Activities of Squabs

The effects of dietary plant polysaccharide supplementation on intestinal enzyme activities are presented in Table 8. In the jejunum, trypsin activity was significantly increased in both the APS and AG groups (*p* < 0.05), and lipase activity was significantly elevated in the AG group (*p* < 0.05), with no significant differences observed in other indicators. Relatively large standard deviations in some enzyme activities may reflect individual variation in intestinal digestive function among early-weaned squabs.

### 3.8. Effects of Dietary Plant Polysaccharides on Intestinal Morphology of Squabs

The effects of dietary plant polysaccharide supplementation on intestinal VH, CD, and the V/C ratio in squabs are presented in Table 9. Compared with the CK group, the AG group exhibited a significantly lower CD and a significantly higher V/C ratio in the duodenum (*p* < 0.01), whereas the APS group showed a significantly increased V/C ratio (*p* < 0.05). In the jejunum, VH and V/C ratios were significantly increased in the AG group (*p* < 0.01). In the ileum, CD was significantly decreased in the AG group (*p* < 0.01), and the V/C ratio was significantly increased in both the GPS and AG groups (*p* < 0.01).

**Figure 1:**
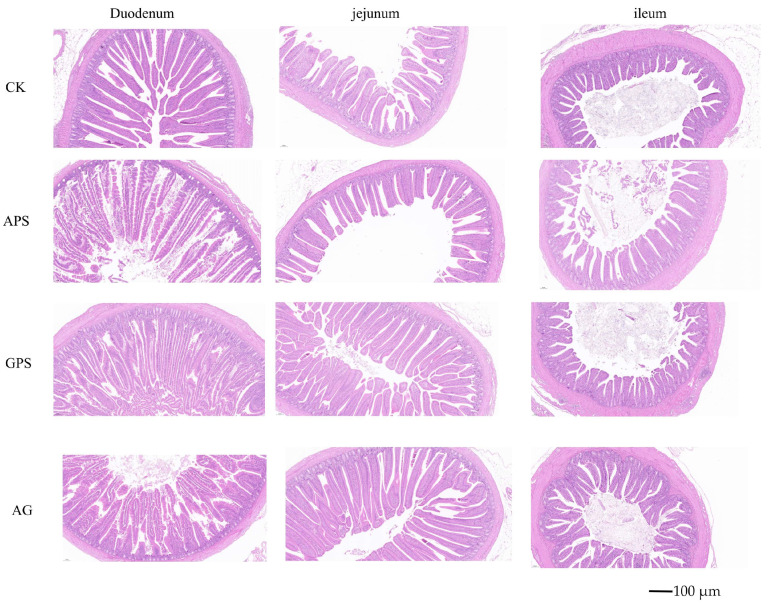
Effects of dietary APS, GPS, and their combination on intestinal histomorphology of squabs. Representative hematoxylin and eosin (HE)-stained sections of the duodenum, jejunum, and ileum are shown. Scale bar = 100 μm.

The effects of dietary plant polysaccharide supplementation on intestinal histomorphology are shown in Figure 1, and the corresponding quantitative measurements of VH, CD, and V/C ratio are summarized in Table 9. In the duodenum, the AG group exhibited a significantly reduced CD and an increased V/C ratio compared with the CK group (*p* < 0.01), while the APS group also showed a significantly increased V/C ratio (*p* < 0.05). In the jejunum, the AG group showed significantly increased VH and V/C ratio compared with the CK group (*p* < 0.01). In the ileum, VH was significantly increased in the GPS and AG groups (*p* < 0.05), whereas CD was significantly reduced and the V/C ratio was significantly increased in the AG group compared with the CK group (*p* < 0.01). These quantitative findings indicate that dietary APS and GPS supplementation, particularly the combined AG treatment, was associated with improved intestinal morphological characteristics. The histological observations shown in Figure 1 were consistent with the quantitative morphometric results presented in Table 9, supporting the interpretation of intestinal structural alterations based on measured parameters rather than subjective observation alone.

### 3.9. Effects of Dietary Supplementation with Plant Polysaccharides on Gut Microbiota of Squabs

#### 3.9.1. Effects of Dietary Supplementation with Plant Polysaccharides on Gut Alpha Diversity of Squabs

Alpha diversity of the gut microbiota in each squab group is summarized in Table 10. The Chao1 index and observed features varied among groups, with relatively higher values in the APS and GPS groups compared with the CK and AG groups. The Shannon and Simpson indices were highest in the APS group, followed by the AG and GPS groups, and lowest in the CK group. In contrast, the dominance index showed the opposite trend, being highest in the CK group and lowest in the APS group. No significant differences were observed in the Pielou evenness index among the groups. These results indicate numerical variations in alpha diversity indices among groups, although no statistically significant differences were observed. The APS group showed numerically higher Shannon and Simpson indices compared with the CK group.

#### 3.9.2. Venn Diagram

**Figure 2:**
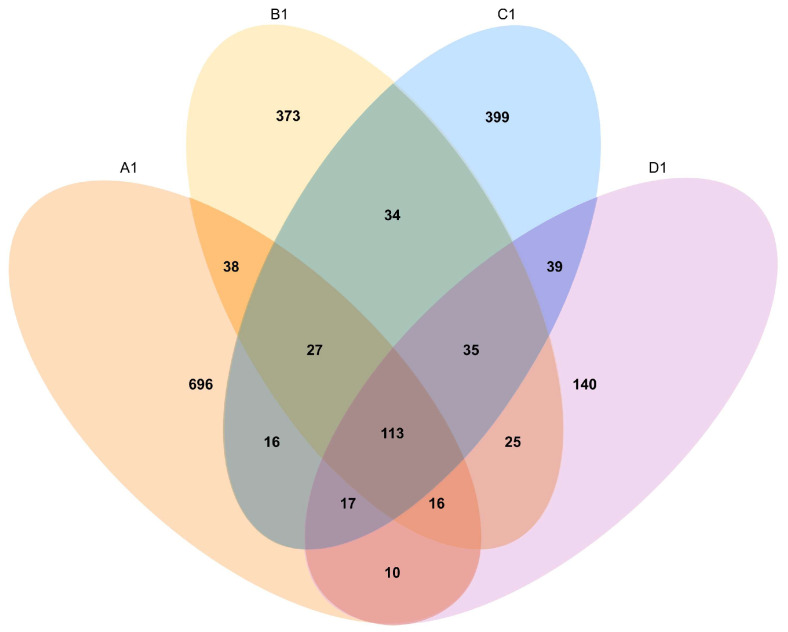
Venn diagram. Numbers indicate the number of ASVs (amplicon sequence variants) shared or unique to each group. The four groups A1, B1, C1 and D1 compared in the figure correspond to the CK group, APS group, GPS group and AG group respectively.

Figure 2 presents a Venn diagram showing shared and unique ASVs among the four groups. A total of 113 ASVs were shared across all groups. The number of unique ASVs was 696 in the CK group, 373 in the APS group, 399 in the GPS group, and 140 in the AG group.

#### 3.9.3. Effects of Dietary Supplementation with Plant Polysaccharides on Intestinal Beta Diversity of Squabs

**Figure 3:**
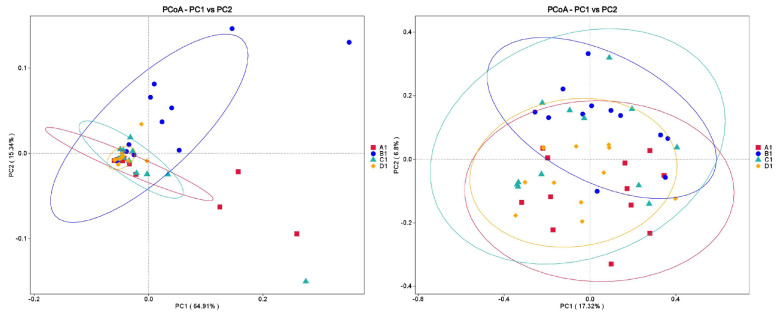
Two−dimensional PCoA diagram (**left**: based on Weighted Unifrac distance; **right**: based on Unweighted Unifrac distance).

Weighted principal coordinate analysis (PCoA) based on PC1 versus PC2 showed that the first and second principal coordinates explained 64.91% and 15.34%, and 17.32% and 6.8% of the total bacterial variation, respectively. The first principal coordinate accounted for a greater proportion of bacterial variation and more effectively distinguished differences among groups (Figure 3).

#### 3.9.4. Effects of Plant Polysaccharides on the Diversity of Intestinal Flora in Squabs

**Figure 4:**
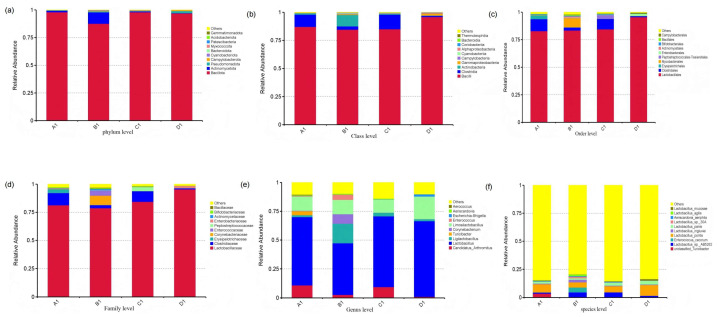
Effects of dietary APS, GPS, and their combination on ileal microbial composition at different taxonomic levels in squabs. The relative abundance of ileal microbiota is shown at the phylum, class, order, family, genus, and species levels. Note: Effects of Plant Polysaccharides on the Diversity of Intestinal Flora in Pigeons. (**a**) Phylum level; (**b**) Class level; (**c**) Order level; (**d**) Family level; (**e**) Genus level; (**f**) Species level.

As shown in Figure 4a, the top 10 phyla of the pigeon intestinal contents are Firmicutes, Actinobacteria, Pseudomonadota, Myxobacteria, Radiobacteria, Cyanobacteria, Campylobacterota, Patescibacteria, Acidobacteria, and Gemmatimonadota. As shown in Figure 4b, the top 10 classes of the pigeon intestinal contents are Bacilli, Clostridia, Actinobacteria, Rickettsia, Alphaproteobacteria, Cyanobacteria, Campylobacteria, Gammaproteobacteria, Bacteroidia, and Thermobacteria. As shown in Figure 4c, the top 10 orders of the pigeon intestinal contents are Lactobacillales, Clostridiales, Erysipelotrichales, Mycobacteriales, Peptostreptococcales-Thermomonadales, Enterobacterales, Actinomycetales, Bifidobacteriales, Bacillales, and Campylobacterales. As shown in Figure 4d, the top 10 families of the pigeon intestinal contents are Lactobacillaceae, Clostridiaceae, Erysipelotrichaceae, Corynebacteriaceae, Enterococcaceae, Peptostreptococcaceae, Enterobacteriaceae, Actinomycetaceae, Bifidobacteriaceae, and Bacillaceae. As shown in Figure 4e, the top 10 genera of the pigeon intestinal contents are *Lactobacillus*, *Lactobacillus rhamnosus*, *Lactobacillus paracasei*, *Corynebacterium*, *Eubacterium*, *Turicibacter*, *Escherichia-Shigella*, *Enterococcus*, *Aeriscardovia*, and *Aerococcus*. As shown in Figure 4f, the top 10 species of the pigeon intestinal contents are *uncultured Turicibacter*, *Lactobacillus* sp. *AB5262*, *Enterococcus cecorum*, *Pontis lactis*, *Lactobacillus ingluviei*, *Lactobacillus panis*, *Lactobacillus* sp. *30A*, *Aeriscardovia aeriphila*, *Lactobacillus crispatus*, and *Lactobacillus mucosae*.

#### 3.9.5. LEfSe Analysis of Ileal Microbiota

**Figure 5:**
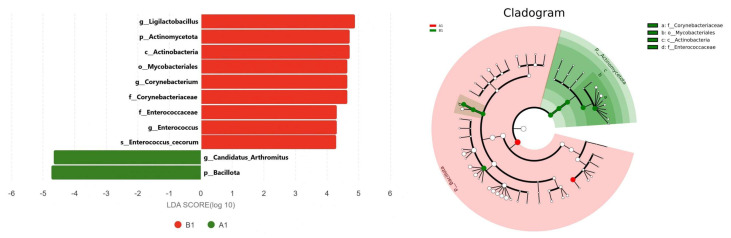
LEfSe analysis of ileal microbiota.

Figure 5 presents the results of LEfSe analysis, which identified species with statistically significant differences among groups. A total of 11 significantly different taxa were identified, mainly enriched in the CK and APS groups. Taxa enriched in the CK group included the phylum Bacillota (p_Bacillota), corresponding to the traditional phylum Firmicutes under the updated bacterial taxonomy nomenclature, and the genus *Candidatus Arthromitus* (g_Candidatus_Arthromitus). Taxa enriched in the APS group included the phylum Actinomycetota (p_Actinomycetota), class Actinobacteria (c_Actinobacteria), order Mycobacteriales (o_Mycobacteriales), family Corynebacteriaceae (f_Corynebacteriaceae) and its genus *Corynebacterium* (g_Corynebacterium), family Enterococcaceae (f_Enterococcaceae) and its genus *Enterococcus* (g_Enterococcus), species *Enterococcus cecorum* (s_Enterococcus_cecorum), and the genus *Ligilactobacillus* (g_Ligilactobacillus).

#### 3.9.6. BugBase Phenotype Prediction

**Figure 6:**
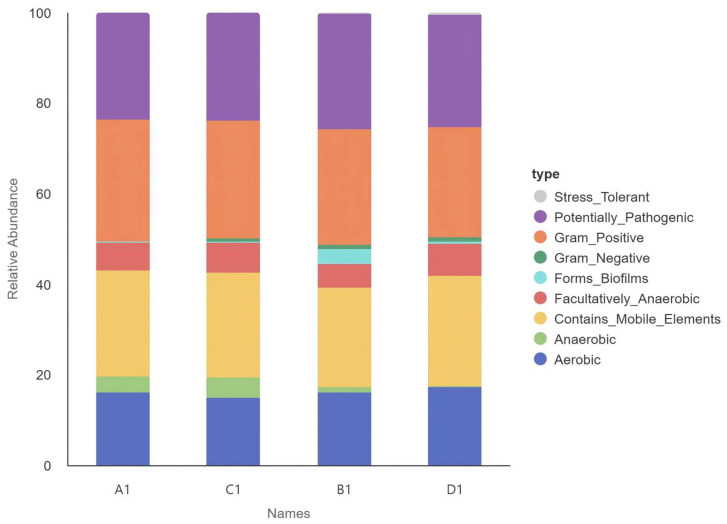
BugBase-predicted microbial phenotypes.

The relative abundance analysis of functional groups within the gut microbiota of pigeons is shown in Figure 6. Potentially pathogenic bacteria (*Potentially_Pathogenic*) and Gram-positive bacteria (*Gram_Positive*) represented the dominant functional groups in all treatments, with their combined abundance exceeding 70% in each group. The abundance of potentially pathogenic bacteria was highest in the AG group, followed by the APS group, and was slightly lower in the CK and GPS groups. The abundance of Gram-positive bacteria remained relatively stable across groups. Aerobic bacteria (*Aerobic*) constituted the third most abundant functional group, with relative abundance ranging from 15% to 18%. The remaining functional groups, including facultatively anaerobic bacteria (*Facultatively_Anaerobic*), bacteria containing mobile elements (*Contains_Mobile_Elements*), anaerobic bacteria (*Anaerobic*), biofilm-forming bacteria (*Forms_Biofilms*), and Gram-negative bacteria (*Gram_Negative*), were present at relatively low abundance, each accounting for less than 10%. In addition, stress-tolerant bacteria (*Stress_Tolerant*) were detected only in trace amounts in the AG group and were absent in the other groups.

#### 3.9.7. PICRUSt Functional Prediction

**Figure 7:**
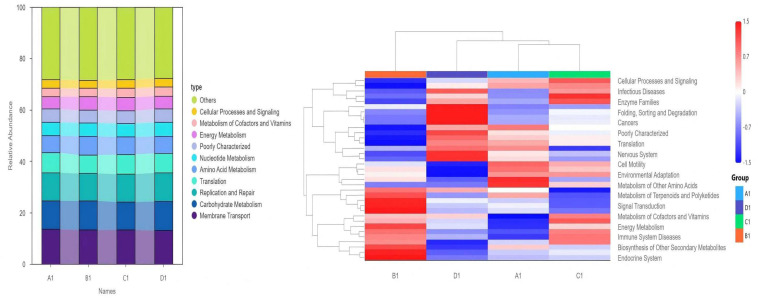
PICRUSt function prediction.

Figure 7 presents the predicted relative abundance distribution of microbial functional pathways based on PICRUSt analysis. Membrane Transport and Carbohydrate Metabolism represented the dominant functional pathways in all groups, together accounting for approximately 30% of the total abundance, with relatively stable distributions across treatments. Other major functional pathways included Replication and Repair, Translation, Amino Acid Metabolism, and Nucleotide Metabolism, each maintaining a relative abundance between 5% and 10% without marked fluctuations among groups. The clustering heatmap further demonstrated differentiation characteristics of functional pathways among treatment groups, with the APS and AG groups clustering together, while the CK and GPS groups formed another cluster. The APS group showed relatively higher predicted abundance of pathways related to Metabolism of Terpenoids and Polyketides and the Endocrine System. The CK group was characterized by higher abundance in pathways including Energy Metabolism and Metabolism of Cofactors and Vitamins. The GPS group exhibited relatively higher abundance in pathways associated with Infectious Diseases and Immune System Diseases. The AG group showed prominent enrichment in pathways related to Cell Motility and Environmental Adaptation.

#### 3.9.8. Genus-Level Co-Occurrence Network Analysis

**Figure 8:**
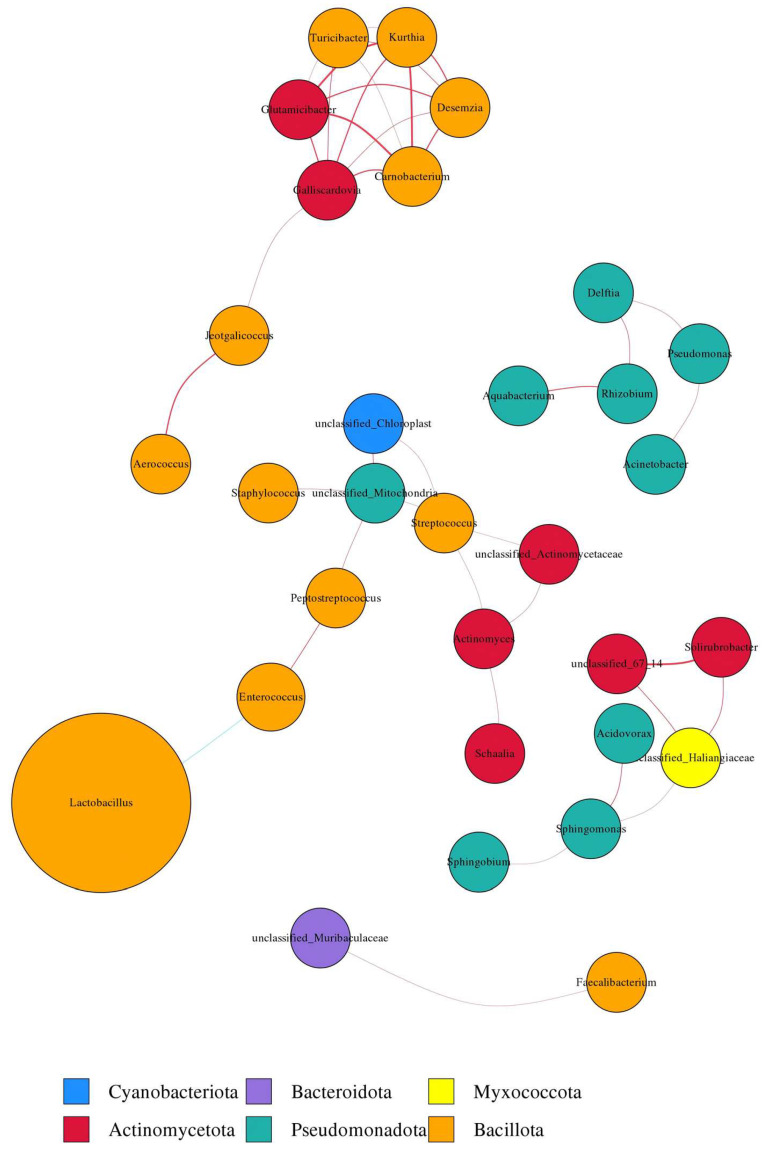
Genus-level cooccurrence network analysis. Note: Different nodes represent different genera, the node size represents the average relative abundance of the genus, and the nodes of the same phylum have the same color (as shown in the legend). The thickness of the connecting line between nodes is positively correlated with the absolute value of the correlation coefficient of species interaction, and the color of the connecting line is positively correlated with the positive and negative correlation (red is positively correlated and blue is negatively correlated).

Following calculation of Spearman correlation coefficients for all samples and construction of the species correlation coefficient matrix, the following filtering criteria were applied: (1) removal of connections with correlation coefficients <0.6; (2) exclusion of node self-connections; and (3) removal of connections with node abundance <0.005%. The resulting network diagram is presented in Figure 8. Genus-level microbial co-occurrence network analysis indicated that taxa belonging to the phylum *Bacillota*, corresponding to the traditional phylum Firmicutes under the updated taxonomy classification, constituted the core structure of the pigeon gut microbial network. Among these taxa, *Lactobacillus* functioned as the key core node and exhibited direct associations with taxa such as Enterococcus. In addition, several genera within *Bacillota*, including *Turicibacter*, *Carnobacterium*, and *Jeotgalicoccus*, clustered together to form a compact functional module. Taxa belonging to the phylum *Actinomycetota*, including *Galliscardovia*, *Actinomyces*, and *Schaalia*, formed another major module within the network. Extensive interactions between this module and Bacillota taxa reflected the coordinated roles of these two phyla in the pigeon gut microecological network. Taxa from the phylum Pseudomonadota, including *Pseudomonas*, *Rhizobium*, and *Sphingomonas*, formed an independent functional subgroup primarily associated with environmental adaptation and substance transport. In addition, a limited number of taxa from the phyla *Cyanobacteriota*, *Bacteroidota*, and *Myxococcota* were incorporated into the network, although their interactions with the core taxa were relatively weak. Overall, the pigeon gut microbial co-occurrence network exhibited clear modular characteristics, with taxa from *Bacillota* and *Actinomycetota* serving as the principal interacting components.

#### 3.9.9. Correlation Analysis of the Top 30 Genera with Detected Indicators

**Figure 9:**
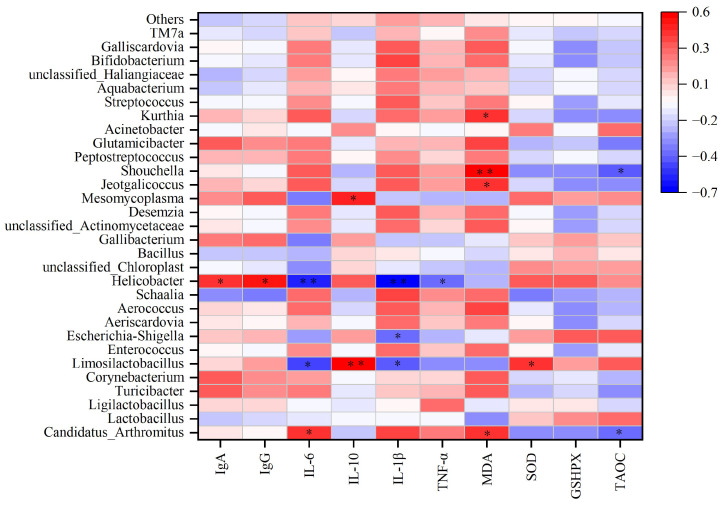
Correlations between the top 30 bacterial genera and serum immune and antioxidant indices. * *p* < 0.05 and ** *p* < 0.01 for the corresponding correlation coefficients.

Pearson correlation coefficients and significance tests were used to determine the correlations between the top 30 bacterial genera at the genus level and serum immune and serum antioxidant parameters. The results are shown in Figure 9. As can be seen from Figure 9, in terms of immune indicators: *Helicobacter* showed a significant positive correlation with IgA and IgG (*p* < 0.05); *Candidatus Arthromitus* showed a significant positive correlation with IL-6 (*p* < 0.05); *Limosilactobacillus* showed a significant negative correlation with IL-6 (*p* < 0.05); *Helicobacter* showed a highly significant negative correlation with IL-6 (*p* < 0.01); *Limosilactobacillus* showed a highly significant positive correlation with IL-10 (*p* < 0.01); *Mesomycoplasma* showed a significant positive correlation with IL-10 (*p* < 0.05); *Limosilactobacillus* and *Escherichia-Shigella* showed significant negative correlations with IL-1β (*p* < 0.05); *Helicobacter* showed a highly significant negative correlation with IL-1β (*p* < 0.01); *Helicobacter* showed a significant negative correlation with TNF-α (*p* < 0.05). In terms of antioxidant indicators, *Candidatus Arthromitus*, *Jeotgalicoccus*, and Kurthia showed significant positive correlations with MDA (*p* < 0.05); *Shouchella* showed a highly significant positive correlation with MDA (*p* < 0.01); *Limosilactobacillus* showed a significant positive correlation with SOD (*p* < 0.05); *Candidatus Arthromitus* and *Shouchella* showed significant negative correlations with T-AOC (*p* < 0.05). Collectively, these results indicate that the top 30 bacterial genera at the genus level are correlated with serum immune and serum antioxidant parameters.

## 4. Discussion

Growth performance represents a key biological indicator reflecting the growth and developmental status as well as metabolic condition of poultry and is primarily characterized by parameters such as body weight gain, growth rate, and feed utilization efficiency during the experimental period [10]. In poultry nutrition and breeding research, growth performance provides an intuitive assessment of the realization of breed genetic potential and effectively reflects the influences of dietary nutrient composition, rearing conditions, and animal health status on growth and development [11]. Numerous studies have demonstrated that plant polysaccharides, including Astragalus polysaccharides and Codonopsis polysaccharides, can increase ADG and feed intake in poultry while improving feed utilization efficiency [12,13,14]. In addition, a meta-analysis further confirmed that Astragalus polysaccharides significantly increase daily gain and reduce the F/G in broilers, indicating stable effects on growth promotion and feed conversion efficiency improvement [15]. The results of the present study suggest that dietary plant polysaccharide supplementation exerted mild numerical increases in growth-related traits in early-weaned squabs, although most parameters did not reach statistical significance. Accordingly, the effects of APS and GPS on growth performance should be interpreted cautiously. The absence of significant differences in several growth indices may be associated with the relatively short experimental duration, supplementation levels, or the intrinsic growth characteristics of squabs. Previous studies have shown that the growth-promoting effects of polysaccharides are influenced by factors including dosage, feeding duration, intestinal microbial responses, and physiological status [16]. Dong et al. reported that polysaccharides promote growth by improving intestinal morphology and antioxidant capacity, although their effects on feed intake and F/G ratio may not always be significant [17]. Moreover, the beneficial effects of plant polysaccharides may not be reflected exclusively through growth performance. Du et al. demonstrated that plant polysaccharides significantly enhanced immune function, increased the levels of immunoglobulins such as IgA, IgG, and IgM, and reduced inflammatory responses through regulation of inflammatory pathways including NF-κB, thereby improving physiological status and supporting growth [18]. In addition, Zhang et al. reported that the antioxidant properties of plant polysaccharides contribute to improved metabolic efficiency and maintenance of physiological homeostasis [19]. In the present study, APS and GPS improved serum immune indices, antioxidant responses, intestinal morphology, digestive enzyme activities, and gut microbiota composition. These findings suggest that the primary effects of plant polysaccharides may involve physiological regulation and alleviation of weaning stress rather than direct stimulation of overall growth performance. Therefore, APS and GPS may contribute to growth regulation and adaptation to early-weaning stress, although their effects on overall growth performance appeared limited under the present experimental conditions.

Body size traits serve as valuable indicators for assessing skeletal development, body conformation, and growth potential in poultry [20,21,22,23,24]. In this study, dietary supplementation with APS, GPS, and their combination improved several body measurement traits, notably breast width, breast depth, keel length, and body length, suggesting a potential role of plant polysaccharides in supporting structural development during early-weaning adaptation. Since most growth-performance indices did not reach statistical significance, these changes should not be interpreted as direct evidence of a strong growth-promoting effect. Rather, improvements in body size traits may be linked to enhanced intestinal development, nutrient absorption, immune regulation, and oxidative–antioxidant balance, which collectively create a more favorable internal environment for body development, particularly under early-weaning stress. Similar effects have been observed by Zhao et al., who reported that supplementation with a probiotic-enzyme complex improved breast width, breast depth, keel length, daily gain, and feed conversion efficiency in broilers [25]. Mechanistically, the observed improvements in body size traits may be associated with enhanced intestinal development and nutrient utilization, as nutritional regulation can promote coordinated bone and muscle growth through improvements in intestinal morphology and absorption capacity [26]. Additionally, the more pronounced responses in breast-related traits compared with shank circumference suggest that APS and GPS exerted stronger effects on trunk development and muscle-related growth than on limb circumference. This interpretation aligns with findings by Qi et al., who reported that breast-related traits are closely correlated with muscle deposition capacity [27]. Overall, the beneficial effects of APS and GPS on body size development appear to result from coordinated regulation of intestinal function, nutrient utilization, physiological adaptation, and muscle-related growth, rather than from a direct impact on body frame expansion.

Serum biochemical, immune, and antioxidant indices represent important parameters for evaluating metabolic status, immune function, oxidative stress, and physiological homeostasis in poultry. Serum proteins, including ALB- and GLB-related components, reflect nutritional metabolism, immune-related protein status, and systemic physiological alterations [28], whereas immunoglobulins, particularly IgG, constitute key components of humoral immunity and participate in immune recognition, pathogen neutralization, and effector immune responses [29]. In the present study, dietary supplementation with APS, GPS, and their combination did not significantly affect basic serum biochemical parameters, suggesting that the applied supplementation levels did not disrupt protein metabolism, lipid metabolism, or hepatic physiological balance in early-weaned squabs. Similar findings have been reported in animals receiving functional feed additives, in which blood biochemical parameters remained relatively stable despite improvements in growth- and health-related traits [30]. These observations indicate that the regulatory effects of APS and GPS may not be primarily reflected through alterations in basic serum biochemical metabolism. Plant-derived bioactive substances are widely recognized as intestinal health enhancers in poultry and may regulate host physiology mainly through modulation of intestinal barrier function, microbial ecology, immune responses, inflammatory balance, and antioxidant defense [31]. Dietary Glycyrrhiza polysaccharides have been reported to improve growth performance and enhance hepatic antioxidant and anti-inflammatory capacity in broiler chickens [32], whereas compound probiotics combined with Astragalus polysaccharides have been shown to regulate serum biochemical indices and fecal microbiota in growing-finishing pigs [33]. In addition, licorice polysaccharides improve immune function in broilers [34], and compound polysaccharides derived from Astragalus and Glycyrrhiza regulate antioxidant function, serum metabolism, and cecal microbiota in broilers [35]. These findings support the view that APS and GPS may improve physiological status primarily through immunomodulatory, anti-inflammatory, and antioxidant mechanisms. Mechanistically, plant polysaccharides may enhance humoral immune defense by promoting immunoglobulin production, reduce excessive inflammatory responses through regulation of immune-related signaling pathways and intestinal microbial balance, and strengthen antioxidant defense by increasing antioxidant enzyme activities and reducing lipid peroxidation [36]. The observed immunomodulatory and antioxidant effects of APS and GPS are likely mediated by well-established signaling pathways. APS is known to activate the chTLR4 pathway in the bursa of Fabricius via an MyD88-independent mechanism, leading to increased sIgA production and to suppress pro-inflammatory cytokines through downregulation of TLR4 and NF-κB transcription [37]. GPS similarly modulates inflammatory responses via the TLRs/NF-κB signaling axis, as confirmed by recent transcriptomic and proteomic analyses in broilers [38]. Furthermore, the antioxidant properties of APS are partly attributable to activation of the Nrf2 pathway, upregulating downstream effectors such as HO-1, TrxR1 and NQO1. GPS likewise enhances total antioxidant capacity and reduces lipid peroxidation through the Nrf2/Keap1 pathway [39]. Both polysaccharides also function as prebiotics, promoting beneficial bacteria such as *Lactobacillus* and *Enterococcus* while suppressing pathogenic taxa, thereby supporting intestinal barrier integrity and modulating the microbial co-occurrence network. Therefore, the stability of serum biochemical parameters together with improvements in immune and antioxidant indices suggests that APS and GPS may contribute to the maintenance of metabolic stability while supporting immune-redox homeostasis and physiological adaptation to early-weaning stress in squabs. However, because inflammatory signaling pathways and antioxidant-related molecular markers were not directly measured in the present study, these mechanistic interpretations remain inferential and require further molecular validation.

Intestinal antioxidant indicators serve as biomarkers for evaluating oxidative stress status and antioxidant defense capacity within the intestine. Plant polysaccharides, recognized as natural antioxidants, have been reported to exert protective effects on intestinal health and may provide novel strategies for disease prevention and control [40]. The present study demonstrated that supplementation with APS, GPS, and their combination (AG) regulated intestinal oxidative–antioxidant status in early-weaned squabs, although the responses differed among intestinal segments. Overall, APS, GPS, and AG increased T-AOC in most intestinal segments, suggesting activation of intestinal antioxidant defense mechanisms. However, these changes did not consistently indicate reduced oxidative damage across all intestinal segments. Increased MDA levels were observed in several intestinal segments, particularly in the APS and AG groups, indicating that lipid peroxidation was not alleviated—and in some cases may have been exacerbated—under these treatment conditions. This apparent discrepancy—simultaneous elevation of both T-AOC and MDA—warrants a more nuanced interpretation. While increased T-AOC and certain antioxidant enzyme activities (e.g., duodenal T-SOD in the APS group) suggest that the intestinal mucosa mounted an antioxidant defense response, the concurrent increase in MDA, a reliable marker of lipid peroxidation and oxidative damage, indicates that oxidative stress was not uniformly relieved. One plausible explanation is that the observed increases in antioxidant enzyme activities represent a compensatory physiological response to an elevated oxidative challenge rather than a direct improvement in oxidative status. Early weaning itself is a known stressor that induces intestinal oxidative stress [41]. Dietary APS and GPS may further stimulate intestinal metabolic activity, microbial fermentation, nutrient utilization, and epithelial remodeling during the adaptation period, thereby increasing the production of reactive oxygen species (ROS). The upregulation of T-AOC and certain antioxidant enzymes could therefore be a reactive, protective attempt by the intestinal mucosa to counteract this heightened oxidative pressure. The failure to reduce MDA levels suggests that this compensatory defense may be insufficient to fully prevent oxidative damage under the current experimental conditions. Alternatively, as mentioned earlier, plant polysaccharide supplementation may promote localized oxidative turnover and transient accumulation of lipid peroxidation products as a consequence of enhanced metabolic activity. These findings differ partially from previous studies reporting reductions in oxidative products following plant polysaccharide supplementation, suggesting that antioxidant responses may depend on animal species, intestinal segment, supplementation strategy, and physiological stress status. Xia Weifeng reported that Astragalus polysaccharides significantly enhanced intestinal antioxidant capacity by increasing antioxidant enzyme activities while reducing oxidative products [42]. Song et al. demonstrated that licorice polysaccharides alleviated oxidative stress through increased antioxidant enzyme activities and decreased MDA levels [43]. In addition, regulation of intestinal antioxidant status by plant polysaccharides may depend not only on direct antioxidant effects but also on modulation of the gut microbiota. Liu et al. found that polysaccharides enhanced antioxidant status by promoting the proliferation of beneficial bacteria, regulating short-chain fatty acid production, and improving intestinal barrier function [44]. Notably, the AG treatment did not consistently produce superior effects compared with single supplementation across all intestinal antioxidant parameters. Although AG exhibited stronger effects in certain serum antioxidant indices, elevated intestinal MDA levels in some segments suggest that combined supplementation may simultaneously enhance metabolic activity and oxidative turnover during adaptation to weaning stress. Importantly, the present data do not support a straightforward conclusion that APS and GPS uniformly alleviate intestinal oxidative stress. Instead, these findings highlight a complex, segment-specific, and context-dependent redox response. Future studies measuring direct ROS levels, mitochondrial function, and specific oxidative damage markers (e.g., protein carbonyls, 8-OHdG) are needed to distinguish between a genuine antioxidant effect and a compensatory elevation of antioxidant enzymes in response to increased oxidative stress. Therefore, the intestinal antioxidant effects of APS and GPS should be interpreted cautiously.

Intestinal digestive enzyme activities are key indicators for assessing digestive capacity, nutrient hydrolysis, and overall intestinal functional status. Enzymes such as amylase, trypsin, and lipase are responsible for the digestion of carbohydrates, proteins, and lipids, respectively, and their activities directly influence nutrient utilization efficiency and growth adaptation in poultry [44]. In the present study, dietary supplementation with APS, GPS, and their combination (AG) affected digestive enzyme activities in the jejunum, with more pronounced changes observed in trypsin and lipase activities. This suggests that plant polysaccharides may enhance the digestive function of early-weaned squabs. These improvements in digestive enzyme activity may be associated with enhanced intestinal morphology and epithelial maturation, which provide a more favorable microenvironment for enzyme activity and nutrient absorption. Consistently, Yang et al. reported that plant polysaccharides improve intestinal structure and function, thereby enhancing digestive enzyme activities and nutrient utilization efficiency [45]. Additionally, the regulatory effects of plant polysaccharides on digestive enzymes may be closely linked to their prebiotic-like properties. Polysaccharides can act as fermentable substrates for intestinal microorganisms, promoting the proliferation of beneficial bacteria and improving the intestinal microbial environment. This, in turn, may indirectly stimulate digestive enzyme secretion or enhance enzyme activity. The relatively stronger response observed in the AG group suggests that combined supplementation with APS and GPS may exert complementary effects on digestive function, likely through coordinated regulation of gut microbiota, microbial metabolites, and intestinal secretory activity. Rong et al. similarly indicated that polysaccharides can enhance digestive enzyme activity and nutrient absorption by modulating microbial metabolites [46]. Overall, APS and GPS appear to improve digestive capacity in early-weaned squabs primarily through coordinated regulation of intestinal morphology, microbial ecology, and digestive enzyme activity, thereby supporting nutrient utilization and adaptation during the early-weaning period.

The relatively large standard deviations observed for some digestive enzyme activities, particularly trypsin and lipase in the jejunum (Table 8), warrant further comment. Such variability likely reflects biological heterogeneity among early-weaned squabs, including individual differences in feed intake, adaptation to weaning stress, and intestinal maturation rates. Additionally, methodological factors such as the timing of sample collection, the inherent variability of enzyme activity assays, and the possible presence of outlying values may have contributed to the observed dispersion. Although significant differences were detected for some enzyme activities, the large inter-individual variability reduced the precision of the estimates. Therefore, these findings should be interpreted cautiously, and studies with larger sample sizes are needed to confirm their robustness.

From a histological perspective, intestinal villi and crypts form the structural basis for nutrient digestion, absorption, epithelial renewal, and barrier maintenance in the avian small intestine [47]. Parameters such as VH, CD, and the V/C ratio are commonly used to evaluate intestinal development and functional maturity. Plant polysaccharides have been reported to regulate intestinal morphology through effects on villus structure, crypt development, and mucosal function [48]. In the present study, dietary supplementation with APS, GPS, and their combination improved intestinal morphological characteristics to varying extents, suggesting that plant polysaccharides may support intestinal development in early-weaned squabs. Although APS increased the duodenal V/C ratio, mild villus disorganization or structural variation was observed in certain intestinal segments, which may be associated with individual variation, local differences among histological sections, or the limited effect of APS alone under early-weaning stress. Accordingly, histological observations were interpreted together with quantitative measurements of VH, CD, and V/C ratio. Overall, the combined APS + GPS treatment produced more consistent improvements in intestinal morphology. Increased VH or V/C ratio generally reflects an enlarged absorptive surface area and improved absorptive capacity, whereas reduced CD may indicate lower epithelial turnover associated with reduced mucosal stress [49]. Similar findings have been reported in animals supplemented with polysaccharides or other functional additives, which increased VH and improved intestinal integrity [50]. Previous studies have also shown that probiotics and plant extracts improve intestinal health by increasing VH, reducing CD, and enhancing epithelial barrier function [51,52]. Mechanistically, these improvements may be associated with reduced inflammatory stimulation, enhanced antioxidant defense, and modulation of gut microbial composition, which collectively contribute to the maintenance of mucosal integrity and epithelial renewal. The relatively stronger response observed in the jejunum may be related to its central role in nutrient digestion and absorption, as this intestinal segment typically possesses well-developed villi and high absorptive capacity and is therefore more responsive to dietary regulation [53]. Therefore, APS and GPS may support intestinal morphological development in early-weaned squabs by promoting mucosal structural development, enhancing absorptive capacity, and maintaining intestinal barrier function, thereby providing a structural basis for improved nutrient utilization and physiological adaptation following weaning.

Intestinal bacterial diversity represents an important indicator of gut microecological health and is closely associated with the growth, development, and intestinal health of pigeons. The results of the present study demonstrated that dietary plant polysaccharide supplementation modulated the structure and function of the gut microbiota in squabs to varying extents. Alpha diversity analysis showed no significant differences among groups. Nevertheless, numerical trends indicated that the APS group had relatively higher Chao1, Shannon, and Simpson indices compared with the CK group, while the AG group showed comparable diversity indices to the CK group. These findings are consistent with previous reports by Lv and Wassie et al., indicating that plant polysaccharides, functioning as prebiotics, improve gut health by promoting the proliferation of beneficial bacteria and increasing microbial diversity [48,50,54]. Notably, the APS group numerically exhibited the highest Shannon and Simpson indices, which may suggest a trend toward greater microbial evenness and community stability, although differences were not statistically significant. Beta diversity analysis further demonstrated clear separation among treatment groups in the PCoA plot, suggesting potential differences in microbial community structure among the treatment groups. This observation agrees with previous studies showing that dietary supplementation with functional polysaccharides influences host metabolism and immune function through modulation of gut microecological composition [55]. Regarding microbial composition, Bacillota, formerly classified as Firmicutes under traditional bacterial taxonomy, and Actinomycetota, formerly classified as Actinobacteria, were identified as the dominant phyla in the ileal microbiota. Notably, *Lactobacillus* occupied a central position within the gut microbial community. This finding is consistent with previous reports indicating that *Lactobacillus* is a dominant genus in the avian intestine and plays an important role in maintaining intestinal barrier function and suppressing pathogenic bacteria [51]. LEfSe analysis further demonstrated enrichment of potentially beneficial bacteria, including *Enterococcus* and *Lactobacillus*, in the APS group, whereas the CK group was enriched with taxa such as Candidatus Arthromitus. These results suggest that plant polysaccharides promote the colonization of beneficial bacteria and optimize microbial community structure. Previous studies have shown that increased abundance of probiotic bacteria is closely associated with enhanced intestinal immunity and reduced inflammatory responses [56].

BugBase phenotype prediction indicated a relatively higher abundance of potentially pathogenic bacteria in the AG group. While this appears inconsistent with the overall improvement in intestinal health, a cautious interpretation is warranted. BugBase infers phenotypes from taxonomic composition using reference databases that may not fully capture pigeon-specific microbial traits, and it does not directly assess pathogenic activity. Moreover, many “potentially pathogenic” taxa can be commensal. Given the concurrent improvements in immune status, digestive enzymes, and intestinal morphology in the AG group, this result likely reflects a shift in microbial community structure rather than genuine pathogenic activation. Similarly, PICRUSt provides inferred functional potential based on 16S rRNA data, not direct evidence of metabolic activity. These findings should be regarded as hypothesis-generating, and future studies (e.g., metagenomics, metabolomics) are needed for validation. Accordingly, both BugBase and PICRUSt results should be interpreted cautiously. Correlation analysis revealed statistical associations between several bacterial genera and serum immune or antioxidant indices. In particular, *Helicobacter* showed positive correlations with IgA and IgG and negative correlations with several pro-inflammatory cytokines. However, correlation does not imply causation, and these findings do not prove that specific bacterial genera directly mediate host physiological changes. Instead, they should be interpreted as hypothesis-generating associations that require experimental validation. Accordingly, *Helicobacter* should be regarded as an associative biomarker rather than a confirmed functional mediator. Collectively, dietary APS and GPS supplementation may contribute to physiological adaptation in early-weaned squabs through modulation of intestinal morphology, digestive enzyme activity, immune status, oxidative–antioxidant balance, and gut microbial community structure. However, these mechanisms remain correlative and inferential. In addition, only one supplementation level was evaluated, and the experimental period was relatively short; therefore, the optimal inclusion levels, dose–response relationships, and long-term effects remain unclear. Further investigations incorporating larger sample sizes, dose–response designs, species- or strain-level microbial identification, metagenomic sequencing, targeted qPCR, culture-based validation, and metabolomic analysis are required to confirm the regulatory mechanisms of APS and GPS.

## 5. Conclusions

In conclusion, dietary APS and GPS supplementation, either alone or in combination, modulated immune status, oxidative–antioxidant balance, intestinal morphology, digestive enzyme activity, and gut microbial composition in early-weaned squabs. Although most growth-performance indices did not reach statistical significance, APS, GPS, and especially the combined APS + GPS treatment showed significant positive effects on several body measurement traits (breast width, breast depth, keel length, and body length), as well as numerical improvements in intestinal morphology and physiological indicators. The combined treatment showed more pronounced effects in several indices, suggesting a potential combined regulatory effect rather than a confirmed synergistic effect. Microbiota analysis indicated changes in ileal microbial composition and associations between some bacterial genera and immune or antioxidant indicators; however, these associations should be interpreted cautiously. Overall, APS and GPS may serve as potential green feed additives to support physiological adaptation and intestinal health in early-weaned squabs. Further studies are needed to confirm the underlying mechanisms and optimal supplementation levels.

## Figures and Tables

**Table 1:** Ingredient composition and nutrient levels of the basal diet for early-weaned squabs (air-dried basis).

Ingredients	Contents (%)
Corn	52.00
Soybean meal	24.50
Soybean oil	1.00
Pea	10.50
Wheat	4.00
Yeast powder	2.00
Stone powder	0.80
Glucose	0.50
Baking soda	0.40
NaCl	0.40
CaHPO_4_	0.60
L-Lys ·HCl	0.24
DL-Met	0.16
Mycotoxin adsorbent	0.10
Premix ^1^	1.80
Wheat bran	1.00
Total	100.00
Nutrient levels ^2^	
ME (MJ/kg)	11.90
CP (%)	18.14
Ca (%)	1.73
P (%)	0.57
Lys (%)	0.77
Met (%)	0.36

Note: ^1^ Premixed feed supplies per kilogram of feed: VA 27,000 IU, VD_3_ 90,000 IU, VE 850 mg, VK_3_ 92 mg, VB_2_ 160 mg, VB_6_ 115 mg, Nicotinamide 1050 mg, calcium D-pantothenate 520 mg, D-biotin 2 mg, folic acid 20 mg, Cu 340 mg, Fe 1700 mg, Mn 1700 mg, Zn 1400 mg; ^2^ Nutrient composition is actually measured value and metabolic energy is calculated value.

**Table 2:** Effects of dietary APS, GPS, and their combination on growth performance of squabs.

Item	CK	APS	GPS	AG	*p*-Value
Average weight (g)					
0 d	371.29 ± 39.25	370.08 ± 41.55	378.54 ± 42.05	371.52 ± 40.63	0.736
7 d	373.67 ± 52.92	381.06 ± 52.64	398.15 ± 54.94	374.85 ± 46.95	0.082
14 d	473.10 ± 67.14	483.96 ± 62.30	496.38 ± 62.34	484.35 ± 52.70	0.329
21 d	488.44 ± 67.11	508.94 ± 62.77	510.08 ± 72.51	514.88 ± 57.63	0.204
28 d	510.40 ± 53.92	526.21 ± 59.34	530.75 ± 55.04	534.71 ± 59.65	0.173
Average daily weight gain (g/d)					
0–28 d	4.97 ± 2.02	5.58 ± 2.04	5.44 ± 2.09	5.83 ± 2.10	0.223
Average Daily Feed Intake (g/d)					
0–28 d	10.43 ± 3.64	11.83 ± 3.72	11.49 ± 3.35	12.24 ± 4.52	0.919
Feed-to-Gain Ratio (F/G) (g/g)					
0–28 d	9.19 ± 4.08	9.06 ± 3.58	8.97 ± 3.68	8.70 ± 4.31	0.940

Note: CK, control group; APS, Astragalus polysaccharide group; GPS, Glycyrrhiza polysaccharide group; AG, combined APS + GPS group.

**Table 3:** Effects of dietary APS, GPS, and their combination on body measurements of squabs.

Item	CK	APS	GPS	AG	*p*-Value
0 d					
Breast width (mm)	34.96 ± 3.19	34.82 ± 2.75	34.77 ± 2.39	33.66 ± 3.53	0.126
Breast depth (mm)	41.90 ± 2.87	41.70 ± 3.19	41.94 ± 2.43	41.30 ± 3.05	0.690
Tibia length (mm)	29.19 ± 2.02	29.77 ± 1.62	29.57 ± 2.14	29.63 ± 1.98	0.511
Keel length (cm)	6.07 ± 0.89	5.97 ± 0.80	5.97 ± 0.49	5.76 ± 0.59	0.189
Body length (cm)	10.46 ± 0.90	10.39 ± 0.81	10.44 ± 0.52	10.23 ± 0.49	0.366
Shank circumference (cm)	2.22 ± 0.10	2.18 ± 0.12	2.20 ± 0.10	2.21 ± 0.12	0.421
28 d					
Breast width (mm)	49.29 ± 2.89 ^B^	52.32 ± 3.88 ^A^	52.27 ± 2.92 ^A^	51.79 ± 3.11 ^A^	<0.001
Breast depth (mm)	57.57 ± 3.67 ^B^	59.68 ± 4.29 ^A^	59.98 ± 3.58 ^A^	59.87 ± 4.10 ^A^	0.008
Tibia length (mm)	32.50 ± 1.71 ^ab^	32.10 ± 2.01 ^b^	33.30 ± 1.83 ^a^	32.54 ± 1.98 ^ab^	0.021
Keel length (cm)	8.43 ± 0.49 ^B^	8.56 ± 0.56 ^AB^	8.71 ± 0.41 ^A^	8.72 ± 0.41 ^A^	0.008
Body length (cm)	11.96 ± 0.52	11.97 ± 0.60	12.04 ± 0.61	12.23 ± 0.45	0.057
Shank circumference (cm)	2.25 ± 0.10	2.28 ± 0.12	2.28 ± 0.08	2.25 ± 0.09	0.234
Total gain from 0 to 28 days					
Breast width (mm)	14.33 ± 4.44 ^B^	17.62 ± 3.79 ^A^	17.50 ± 4.15 ^A^	18.10 ± 4.26 ^A^	<0.001
Breast depth (mm)	15.67 ± 4.38 ^B^	18.09 ± 3.94 ^A^	18.00 ± 4.11 ^A^	18.56 ± 4.42 ^A^	0.004
Tibia length (mm)	3.31 ± 2.64 ^ab^	2.33 ± 2.08 ^b^	3.73 ± 2.53 ^a^	2.84 ± 2.23 ^ab^	0.029
Keel length (cm)	2.36 ± 0.94 ^Bc^	2.61 ± 0.93 ^ABbc^	2.73 ± 0.65 ^AaBb^	2.97 ± 0.75 ^Aa^	0.004
Body length (cm)	1.50 ± 1.06 ^b^	1.59 ± 0.90 ^b^	1.59 ± 0.84 ^b^	1.99 ± 0.59 ^a^	0.028
Shank circumference (cm)	0.09 ± 0.08	0.12 ± 0.10	0.10 ± 0.09	0.09 ± 0.08	0.461

Note: CK, control group; APS, Astragalus polysaccharide group; GPS, Glycyrrhiza polysaccharide group; AG, combined APS + GPS group. Within the same row, values with different lowercase letters indicate a significant difference (*p* < 0.05), and values with different uppercase letters indicate a highly significant difference (*p* < 0.01).

**Table 4:** Effects of dietary APS, GPS, and their combination on serum biochemical indices of squabs.

Item	CK	APS	GPS	AG	*p*-Value
TP (g/L)	27.53 ± 4.36	27.05 ± 1.75	29.50 ± 5.91	27.13 ± 1.94	0.381
ALB (g/L)	10.47 ± 1.30	10.20 ± 1.65	10.65 ± 1.26	10.34 ± 1.31	0.873
TC (mmol/L)	7.05 ± 1.31	6.70 ± 1.24	6.56 ± 0.76	6.76 ± 1.25	0.764
TG (mmol/L)	1.80 ± 0.67	1.68 ± 1.14	1.47 ± 0.28	1.57 ± 0.47	0.708
GLB (g/L)	17.06 ± 3.96	16.85 ± 1.61	18.85 ± 5.95	16.79 ± 2.04	0.501
HDL (mmol/L)	6.07 ± 0.83	5.80 ± 1.02	5.86 ± 0.68	6.00 ± 1.05	0.880
LDL (mmol/L)	2.76 ± 0.84	2.47 ± 0.81	2.30 ± 0.39	2.50 ± 0.76	0.488
ALT (U/L)	19.25 ± 3.96	23.95 ± 8.06	20.09 ± 6.60	19.09 ± 3.13	0.150
AST (U/L)	89.76 ± 19.02	113.47 ± 45.50	95.53 ± 33.77	98.73 ± 39.94	0.425

Note: CK, control group; APS, Astragalus polysaccharide group; GPS, Glycyrrhiza polysaccharide group; AG, combined APS + GPS group. TP, total protein; ALB, albumin; TC, total cholesterol; TG, triglyceride; GLB, globulin; HDL, high-density lipoprotein; LDL, low-density lipoprotein; ALT, alanine aminotransferase; AST, aspartate aminotransferase.

**Table 5:** Effects of dietary APS, GPS, and their combination on serum immune indices of squabs.

Item	CK	APS	GPS	AG	*p*-Value
IgA (pg/mL)	2.16 ± 0.18 ^b^	2.25 ± 0.13 ^ab^	2.31 ± 0.22 ^a^	2.34 ± 0.10 ^a^	0.045
IgG (ug/mL)	3.91 ± 0.21 ^Bb^	4.14 ± 0.20 ^AaB^	4.18 ± 0.29 ^Aa^	4.28 ± 0.18 ^Aa^	0.002
IgM (ug/mL)	1.49 ± 0.23	1.60 ± 0.19	1.64 ± 0.29	1.65 ± 0.21	0.345
IL-6 (pg/mL)	141.34 ± 11.48 ^Aa^	131.89 ± 6.25 ^AaB^	120.42 ± 8.05 ^Bb^	98.15 ± 19.61 ^Cc^	<0.001
IL-10 (pg/mL)	11.82 ± 1.15 ^Cc^	13.41 ± 2.17 ^BbCc^	14.87 ± 2.15 ^AaBb^	15.89 ± 2.05 ^Aa^	<0.001
IL-1β (pg/mL)	20.51 ± 1.60 ^A^	19.13 ± 1.47 ^A^	16.85 ± 2.16 ^B^	14.36 ± 2.19 ^C^	<0.001
TNF-α (pg/mL)	59.40 ± 7.99 ^Aa^	54.17 ± 3.92 ^ABb^	48.65 ± 5.30 ^BCc^	42.86 ± 3.78 ^Cd^	<0.001

Note: CK, control group; APS, Astragalus polysaccharide group; GPS, Glycyrrhiza polysaccharide group; AG, combined APS + GPS group. Within the same row, values with different lowercase letters indicate a significant difference (*p* < 0.05), and values with different uppercase letters indicate a highly significant difference (*p* < 0.01). IgA, immunoglobulin A; IgG, immunoglobulin G; IgM, immunoglobulin M; IL, interleukin; TNF-α, tumor necrosis factor-α.

**Table 6:** Effects of dietary APS, GPS, and their combination on serum antioxidant indices of squabs.

Item	CK	APS	GPS	AG	*p*-Value
T-AOC (U/mL)	7.09 ± 0.57 ^Cc^	8.29 ± 1.00 ^Bb^	9.09 ± 1.11 ^AaB^	9.65 ± 1.02 ^Aa^	<0.001
T-SOD (U/mL)	71.77 ± 3.87 ^Cd^	76.22 ± 4.41 ^BCc^	80.21 ± 5.75 ^Bb^	89.44 ± 4.58 ^Aa^	<0.001
GSH-Px (U/mL)	140.56 ± 10.68 ^bC^	154.88 ± 18.88 ^BbC^	171.80 ± 7.23 ^AaB^	184.06 ± 28.11 ^Aa^	<0.001
MDA (nmol/mL)	3.11 ± 0.72 ^Aa^	2.76 ± 0.56 ^Aab^	2.58 ± 0.75 ^ABb^	2.04 ± 0.27 ^Bc^	<0.001

Note: CK, control group; APS, Astragalus polysaccharide group; GPS, Glycyrrhiza polysaccharide group; AG, combined APS + GPS group. Within the same row, values with different lowercase letters indicate a significant difference (*p* < 0.05), and values with different uppercase letters indicate a highly significant difference (*p* < 0.01). T-AOC, total antioxidant capacity; T-SOD, total superoxide dismutase; GSH-Px, glutathione peroxidase; MDA, malondialdehyde.

**Table 7:** Effects of dietary APS, GPS, and their combination on intestinal antioxidant indices of squabs.

Item	CK	APS	GPS	AG	*p*-Value
Duodenum					
T-AOC (U/mL)	0.10 ± 0.01 ^B^	0.14 ± 0.02 ^A^	0.14 ± 0.02 ^A^	0.15 ± 0.03 ^A^	<0.001
T-SOD (U/mL)	276.55 ± 19.74 ^b^	314.63 ± 27.69 ^a^	295.92 ± 30.22 ^ab^	284.29 ± 34.58 ^b^	0.013
GSH-Px (U/mL)	11.75 ± 2.85	13.61 ± 2.22	15.07 ± 4.34	14.04 ± 2.35	0.075
MDA (nmol/mL)	0.50 ± 0.07 ^Cc^	0.71 ± 0.12 ^AaBb^	0.62 ± 0.15 ^BbC^	0.80 ± 0.15 ^Aa^	<0.001
Jejunum					
T-AOC (U/mL)	0.09 ± 0.01 ^B^	0.13 ± 0.03 ^A^	0.13 ± 0.02 ^A^	0.12 ± 0.02 ^A^	<0.001
T-SOD (U/mL)	298.44 ± 21.68	292.76 ± 34.85	287.49 ± 40.66	279.13 ± 27.49	0.506
GSH-Px (U/mL)	20.07 ± 8.95	13.29 ± 4.12	18.75 ± 6.97	17.41 ± 8.02	0.133
MDA (nmol/mL)	0.44 ± 0.07 ^Cc^	0.58 ± 0.16 ^BbC^	0.64 ± 0.13 ^AaBb^	0.75 ± 0.15 ^Aa^	<0.001
Ileum					
T-AOC (U/mL)	0.09 ± 0.02 ^B^	0.12 ± 0.03 ^A^	0.10 ± 0.01 ^AB^	0.11 ± 0.02 ^A^	0.007
T-SOD (U/mL)	294.40 ± 31.32	300.86 ± 33.52	288.64 ± 20.32	290.02 ± 17.78	0.676
GSH-Px (U/mL)	28.33 ± 3.32 ^a^	23.29 ± 5.20 ^b^	27.39 ± 4.17 ^a^	28.42 ± 4.47 ^a^	0.018
MDA (nmol/mL)	0.49 ± 0.10 ^Bc^	0.63 ± 0.14 ^Bb^	0.59 ± 0.14 ^Bbc^	0.94 ± 0.17 ^Aa^	<0.001

Note: CK, control group; APS, Astragalus polysaccharide group; GPS, Glycyrrhiza polysaccharide group; AG, combined APS + GPS group. Within the same row, values with different lowercase letters indicate a significant difference (*p* < 0.05), and values with different uppercase letters indicate a highly significant difference (*p* < 0.01). T-AOC, total antioxidant capacity; T-SOD, total superoxide dismutase; GSH-Px, glutathione peroxidase; MDA, malondialdehyde.

**Table 8:** Effects of dietary APS, GPS, and their combination on intestinal enzyme activities of squabs.

Item	CK	APS	GPS	AG	*p*-Value
Duodenum					
Trypsin	1757.20 ± 1434.69	2763.05 ± 1743.90	2699.94 ± 2085.12	3690.15 ± 2905.97	0.187
Chymotrypsin	1.79 ± 0.45	1.99 ± 0.69	1.94 ± 0.54	2.04 ± 0.59	0.726
Lipase	237.63 ± 169.68	355.01 ± 220.61	317.44 ± 160.53	355.85 ± 251.95	0.455
Amylase	17.27 ± 14.33	27.02 ± 17.63	35.74 ± 27.37	38.16 ± 31.21	0.139
Jejunum					
Trypsin	3009.92 ± 2200.63 ^b^	5825.32 ± 2695.06 ^a^	3947.98 ± 2571.65 ^ab^	5808.95 ± 3301.05 ^a^	0.033
Chymotrypsin	1.10 ± 0.56	1.33 ± 0.56	1.33 ± 0.32	1.26 ± 0.28	0.539
Lipase	181.49 ± 97.55 ^b^	285.10 ± 193.31 ^ab^	175.81 ± 66.80 ^b^	319.00 ± 148.69 ^a^	0.025
Amylase	95.55 ± 27.41	84.49 ± 34.83	101.13 ± 42.74	90.09 ± 37.72	0.704

Note: CK, control group; APS, Astragalus polysaccharide group; GPS, Glycyrrhiza polysaccharide group; AG, combined APS + GPS group. Within the same row, values with different lowercase letters indicate a significant difference (*p* < 0.05).

**Table 9:** Effects of plant polysaccharides on intestinal VH, CD, and V/C ratio in squabs.

Item	CK	APS	GPS	AG	*p*-Value
Duodenum					
Villus height (μm)	1005.73 ± 113.64	1022.95 ± 120.09	1012.19 ± 85.57	1037.00 ± 86.15	0.887
Crypt depth (μm)	109.65 ± 11.16 ^A^	102.31 ± 11.66 ^A^	105.59 ± 8.87 ^A^	91.03 ± 6.84 ^B^	<0.001
V/C ratio	9.19 ± 0.67 ^Bc^	10.06 ± 1.20 ^Bb^	9.63 ± 0.94 ^Bbc^	11.42 ± 0.97 ^Aa^	<0.001
Jejunum					
Villus height (μm)	616.46 ± 83.21 ^B^	649.93 ± 58.55 ^B^	631.68 ± 96.73 ^B^	748.91 ± 45.76 ^A^	<0.001
Crypt depth (μm)	98.54 ± 7.98	92.54 ± 17.75	93.61 ± 17.29	87.30 ± 16.00	0.362
V/C ratio	6.28 ± 0.82 ^B^	7.27 ± 1.53 ^B^	6.84 ± 0.90 ^B^	8.87 ± 1.84 ^A^	<0.001
Ileum					
Villus height (μm)	254.07 ± 67.56 ^b^	294.21 ± 59.10 ^ab^	309.53 ± 56.08 ^a^	326.96 ± 67.16 ^a^	0.043
Crypt depth (μm)	82.74 ± 5.15 ^Aa^	76.32 ± 9.93 ^ABb^	76.74 ± 6.90 ^ABb^	71.31 ± 5.32 ^Bb^	0.004
V/C ratio	3.08 ± 0.80 ^Bb^	3.90 ± 0.90 ^AaB^	4.09 ± 0.97 ^Aa^	4.58 ± 0.83 ^Aa^	0.001

Note: CK, control group; APS, Astragalus polysaccharide group; GPS, Glycyrrhiza polysaccharide group; AG, combined APS + GPS group. Within the same row, values with different lowercase letters indicate a significant difference (*p* < 0.05), and values with different uppercase letters indicate a highly significant difference (*p* < 0.01). VH, villus height; CD, crypt depth; V/C ratio, villus height-to-crypt depth ratio.

**Table 10:** Effects of plant polysaccharides on alpha diversity of squabs.

Items	CK	APS	GPS	AG	*p*-Value
Chao1	80.84 ± 29.88	104.37 ± 26.53	98.32 ± 49.32	79.76 ± 15.50	0.208
Observed features	79.27 ± 29.99	98.09 ± 25.85	94.36 ± 46.81	78.00 ± 14.16	0.331
Dominance	0.26 ± 0.08	0.22 ± 0.08	0.24 ± 0.11	0.25 ± 0.11	0.820
Shannon	2.98 ± 0.71	3.18 ± 0.38	3.01 ± 0.71	3.12 ± 0.57	0.856
Pielou e	0.48 ± 0.08	0.49 ± 0.07	0.47 ± 0.11	0.50 ± 0.09	0.909
Simpson	0.74 ± 0.08	0.78 ± 0.08	0.76 ± 0.11	0.75 ± 0.11	0.820

## Data Availability

The data that support the findings of this study are available from the corresponding author upon reasonable request.

## Abbreviations

The following abbreviations are used in this manuscript:

APS	Astragalus polysaccharide
GPS	Glycyrrhiza polysaccharide
AG	APS + GPS combined group
CK	Control group
ADG	Average daily gain
ADFI	Average daily feed intake
F/G	Feed-to-gain ratio
TP	Total protein
ALB	Albumin
GLB	Globulin
TC	Total cholesterol
TG	Triglyceride
HDL	High-density lipoprotein
LDL	Low-density lipoprotein
ALT	Alanine transaminase
AST	Aspartate transaminase
IgA	Immunoglobulin A
IgG	Immunoglobulin G
IgM	Immunoglobulin M
IL-6	Interleukin-6
IL-1β	Interleukin-1β
IL-10	Interleukin-10
TNF-α	Tumor necrosis factor-α
T-AOC	Total antioxidant capacity
T-SOD	Total superoxide dismutase
GSH-Px	Glutathione peroxidase
MDA	Malondialdehyde
V/C	Villus height/Crypt depth ratio
HE	Hematoxylin-eosin
PCoA	Principal coordinate analysis
LEfSe	Linear discriminant analysis Effect Size
PICRUSt	Phylogenetic Investigation of Communities by Reconstruction of Unobserved States
ME	Metabolic energy
CP	Crude protein
